# Suppression of Metastasis of Colon Cancer to Liver in Mouse Models by Pretreatment with Extracellular Vesicles Derived from *Nanog*-Overexpressing Colon-26 Cancer Cells

**DOI:** 10.3390/ijms252312794

**Published:** 2024-11-28

**Authors:** Takuya Henmi, Hideaki Matsuoka, Noa Katayama, Mikako Saito

**Affiliations:** 1 Department of Biotechnology and Life Science, Tokyo University of Agriculture and Technology, 2-24-16, Naka-cho, Koganei 184-8588, Tokyo, Japan; 2 Bioresource Laboratories, Tokyo University of Agriculture and Technology, 2-24-16, Naka-cho, Koganei 184-8588, Tokyo, Japan

**Keywords:** colon-26, *Nanog*, metastasis, macrophage, phagocytosis

## Abstract

It has been demonstrated that cancer cells that have survived cancer treatment may be more malignant than the original cancer cells. These cells are considered the main cause of metastasis in prognosis. A *Nanog*-overexpressing colon-26 (*Nanog*^+^colon26) was generated to obtain such a malignant cancer cell model, which was confirmed by enhancement of metastatic potential by in vivo tests using mice. Extracellular vesicles (EVs) secreted from *Nanog*^+^colon26 cells (*Nanog*^+^colon26EVs) were administered to mice three times per week for three weeks. Subsequently, *Nanog*^+^colon26 cells were administered, and metastatic colonies were analyzed two weeks later. The results demonstrated that the administration of EVs suppressed metastasis. *Nanog*^+^colon26EVs enhanced phagocytic activity and M1 marker CD80 of a macrophage cell line J774.1. These suggested the enforcement of tumor-suppressive properties of macrophages and their contribution to the in vivo suppression of metastasis. Small RNA sequencing was conducted to identify *Nanog*-dependent miRNAs that exhibited significant changes (Fc ≥ 1.5 or Fc ≤ 1/1.5; *p* < 0.05) in *Nanog*^+^colon26EVs relative to colon26EVs. Nine miRNAs (up-regulated: four, down-regulated: five) were identified, and 623 genes were predicted to be their target genes. Of the 623 genes identified, nine genes were predicted to be highly relevant to macrophage functions such as phagocytosis.

## 1. Introduction

While anticancer drugs and radiation treatments are effective in killing cancer cells, some cells are able to evade these treatments. It can be postulated that those cells should have acquired high resistance to drugs [1,2,3] and radiation [4,5] and thus be regarded as malignant cancer cells. It is believed that these cells are the primary causes of recurrence and metastasis and present a significant challenge in cancer treatment. Therefore, a malignant cancer cell model is thought to be necessary for the development of therapeutic methods.

Cancer stem cells (CSCs) have been implicated in cancer malignant transformation [6,7,8]. CSCs are a small subpopulation of cancer cells that are drug-resistant and support tumor recurrence and distant metastasis [9,10,11]. However, CSCs have different definitions and properties depending on the type of cancer, and it has been difficult to obtain stable cells with stable performance that can be used for metastasis inhibition studies. Therefore, we focused on the anaplastic property, one of the characteristics of CSCs, and intended to develop a malignant cancer cell line by lowering the differentiation level.

*Nanog* was selected as an inducer of an anaplastic state. Based on previous studies on the anaplastic state of mouse embryonic stem cells, *Nanog* was thought to be the best compared to other factors such as *Oct3/4*, *Sox2*, and *Cdx2*. Positive correlations between the expression level of *Nanog* and malignancy were observed in 14 types of cancer, including breast cancer, colon cancer, embryonic carcinoma, liver cancer, and skin cancer (melanoma) [12,13,14]. We selected murine melanoma as the first target cancer, because several melanoma cell lines with different metastatic potential (B16BL6, B16F10, and B16F1) had been established, and stable cell lines were available. In addition, these cells tend to metastasize to the liver and lungs, and metastatic colonies are expected to be easy to count and analyze as black masses. *Nanog*-overexpressing melanoma enhanced metastatic potential [15]. We thought it enough to use those cell lines for our experimental purpose, although the resistance to anticancer drugs was not necessarily enhanced.

The next issue to consider is using EVs to prevent the metastasis of malignant cancer cells. Formerly, cancer cell-derived EVs were introduced into immune cells such as dendritic cells, and then these dendritic cells demonstrated cancer immunotherapy [16,17,18]. EVs were incorporated into bone marrow-derived dendritic cells in vitro, and the dendritic cells demonstrated excellent therapeutic effects [16]. The introduction of cells into the body, however, carries the risk of side effects. By contrast, the introduction of cell-free EVs does not have such a risk. The cell-free EVs method was adopted in recent therapies for COVID-19 [19,20]. EVs are small in size and are thought to be incorporated not only into immune cells but also into a significantly larger number of normal cells. Therefore, if EVs with properties that activate normal cells and increase their resistance to foreign cell invasion are introduced, the entire cells in the whole body will be able to exert strong resistance to cancer metastasis. The EVs method, which maintains immune function while reducing the risk of side effects, is expected to reduce the dosage of anticancer drugs and alleviate their side effects when used in combination with conventional anticancer drug treatments. Therefore, vaccines utilizing EVs derived from cancer cells have recently garnered significant interest [21,22,23].

Since EVs comprise a multitude of components [24,25], it is important to evaluate their performance. For example, it has been observed that EVs contain components with tumor-suppressing effects, such as interleukin-12 (IL-12) [26], and components with tumor-promoting effects, such as *Tgf-β* [27]. In fact, overexpression of *IL-12* and knockdown of *Tgf-β* showed antitumor effects [28]. Components that contribute to tumor suppression in one cancer, such as miR-205 [29], may have tumor-promoting effects in another cancer. Therefore, it is necessary to establish a method to accurately evaluate the performance of EVs according to differences in cancer type and disease progression stage.

Initially, EVs were prepared from *Nanog*-overexpressing melanoma. The EVs were expected to promote metastasis. If as expected, we would have analyzed the influence of *Nanog* to find clues to inhibit it. Contrary to our expectations, however, the EVs showed metastasis-suppressing effects [30], suggesting that *Nanog*^+^F10EVs are an autologous vaccine candidate that suppresses the metastasis of *Nanog*^+^F10. We also obtained results suggesting that immune cells, particularly macrophages, are involved in the metastasis-suppressing effect [30,31]. Therefore, we decided to investigate whether this phenomenon could be seen in other cancers.

Mouse colon cancer was selected as the second target. The reason for its selection was simply that, according to future predictions by the Global Cancer Observatory, Cancer Tomorrow [32], colon and/or rectal cancer has the highest morbidity and mortality rates of all cancers. Colorectal cancer is prone to metastasis to the liver and lungs, and colon cancer, in particular, has a high tendency to metastasize to the liver [33]. It is believed that suppressing liver metastasis will improve mortality. Depending on the stage of cancer progression, surgery, chemotherapy, radiation therapy, or a combination of these is performed. Regarding immunotherapy, clinical trial results have shown that the immune checkpoint inhibitor pembrolizumab is effective in mismatch repair–deficient stage IV colon cancer [34]. However, the effectiveness of other immunotherapies has not been proven. There is also a large problem of side effects.

On the other hand, regarding the involvement of *Nanog* in colorectal cancer, for example, it has been reported that the expression of stem cell markers, including *Nanog*, was reduced when furin, which is involved in calcium transport, was inhibited in colorectal cancer [35]. Conversely, it is presumed that an increase in the expression level of *Nanog* enhances the stemness of cancer cells and increases their malignancy. In the case of colorectal cancer, it has been reported that overexpression of *Nanog* increases the expression level of dormancy via the transcription factors p21 and p27 [36]. An increase in the dormancy state means an increase in resistance to anticancer drugs.

Based on the above considerations, this study aims to investigate whether overexpression of *Nanog* increases the malignancy of colon26 cells and whether EVs derived from *Nanog*-overexpressing colon cancer cells can suppress cancer metastasis, and to analyze the involvement of immune cells, especially macrophages, in the mechanism.

## 2. Results

### 2.1. Properties of Nanog-Overexpressing Colon-26 (Nanog^+^colon26) Cells

**Figure 1:**
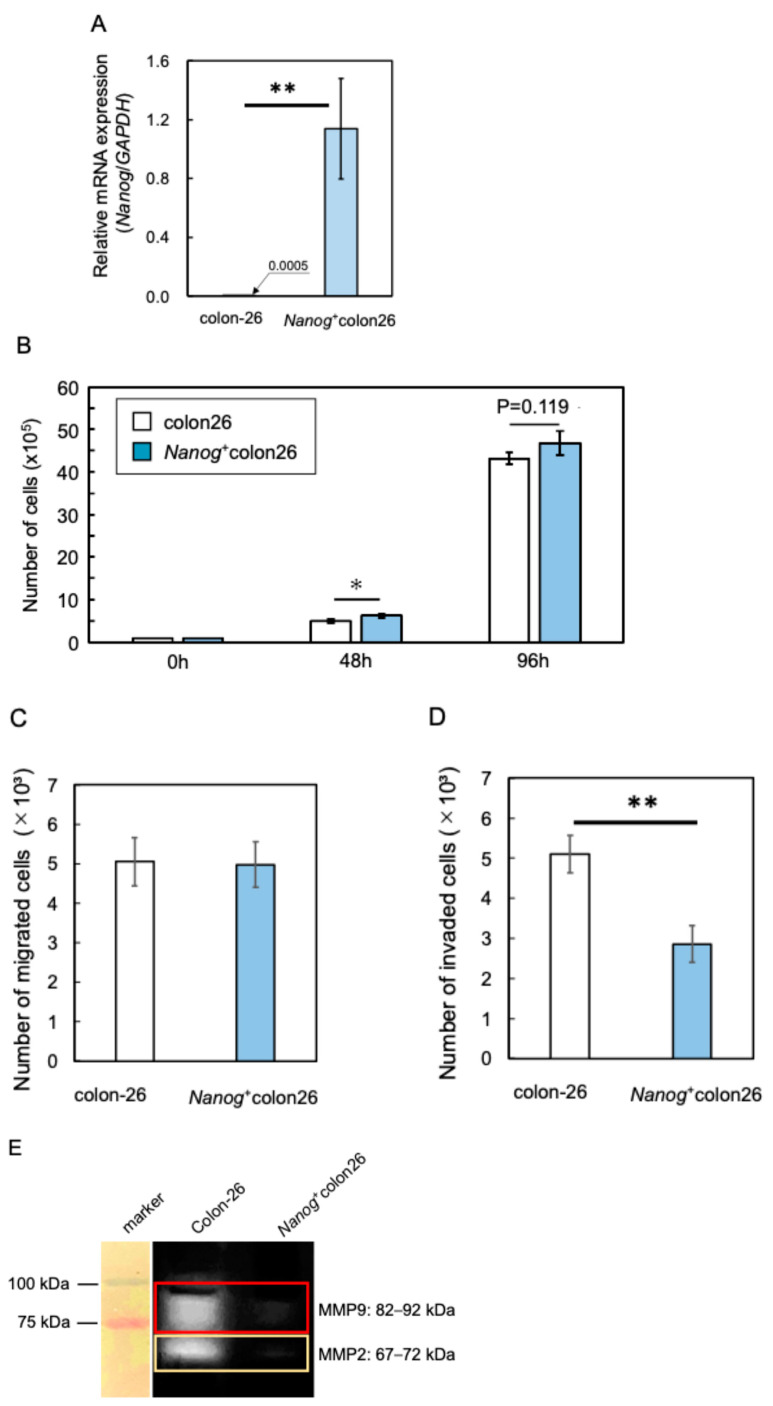
Properties of *Nanog*⁺colon26 cells. (**A**) Relative expression of mRNA of *Nanog*; mean ± SD for *n* = 3. (**B**) Proliferation activity; mean ± SD for *n* = 3. (**C**) Migration activity; mean ± SD for *n* = 3. (**D**) Invasion activity; mean ± SD for *n* = 3. (**E**) Matrix metalloproteinase activity; mean ± SD for *n* = 3. **: *p* < 0.01; *: *p* < 0.05.

**Figure 2:**
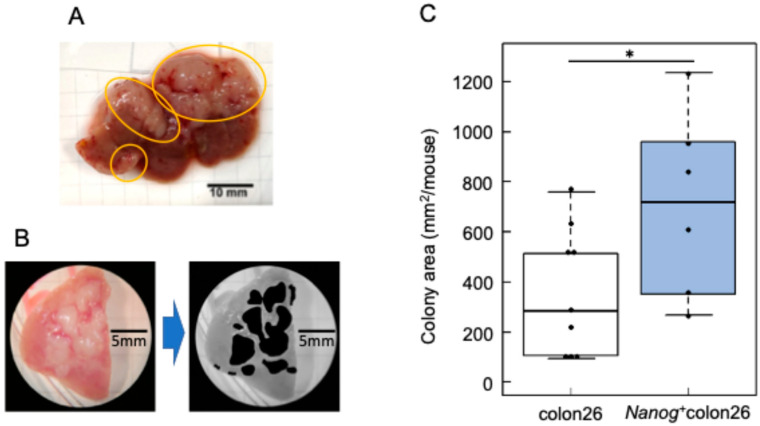
Metastatic properties of *Nanog*⁺colon26 cells. (**A**) Colon cancer colonies metastasize to the liver. Colony: circled by yellow lines. (**B**) Quantification of metastasis by adding up the area of colonies by using ImageJ. (**C**) Colony area per mouse. colon-26: *n* = 7; *Nanog*^+^colon26: *n* = 6; *: *p* < 0.05.

A Nanog overexpression vector was introduced into colon-26 cells to generate a *Nanog*^+^colon26 cell line. The introduction of *Nanog* was confirmed by qPCR (Figure 1A). The *Nanog*^+^colon26 cells were tested for metastasis-related in vitro properties. As compared to colon-26, the proliferation rate was higher as expected (Figure 1B). However, the migration activity was unchanged (Figure 1C), and the invasion activity was lower, as determined by Transwell^TM^ assay (Figure 1D). In addition, matrix metalloproteinases such as MMP2 and MMP9 were less active, suggesting low activity of invasion by the degradation of cell stroma (Figure 1E). In contrast, metastasis to the liver was extremely prominent. White, flat colonies were formed (Figure 2A), and the area of these colonies was added up per mouse to determine the quantity of metastasis (Figure 2B). The metastatic potential of *Nanog*^+^colon26 was significantly higher than that of colon-26 (Figure 2C). In the case of colon-26, *Nanog* overexpression also enhanced the metastatic potential. However, unlike melanoma, in vitro properties of *Nanog*^+^colon26 cells were not so significantly more malignant than colon-26.

### 2.2. Metastasis Suppression Effect of Nanog^+^colon26EVs

**Figure 3:**
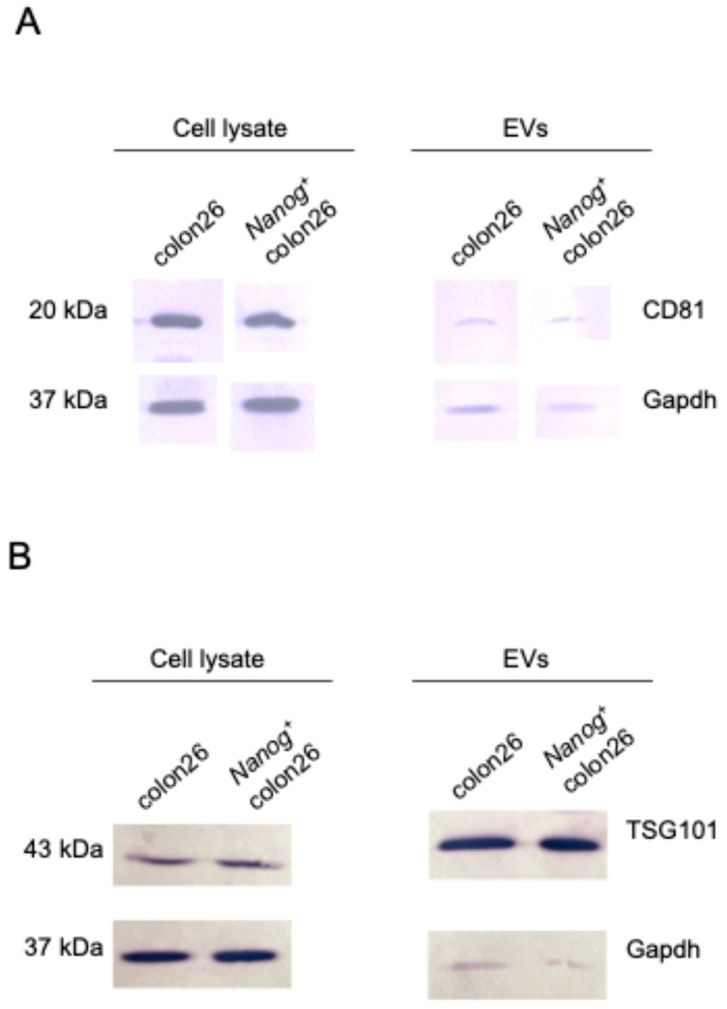

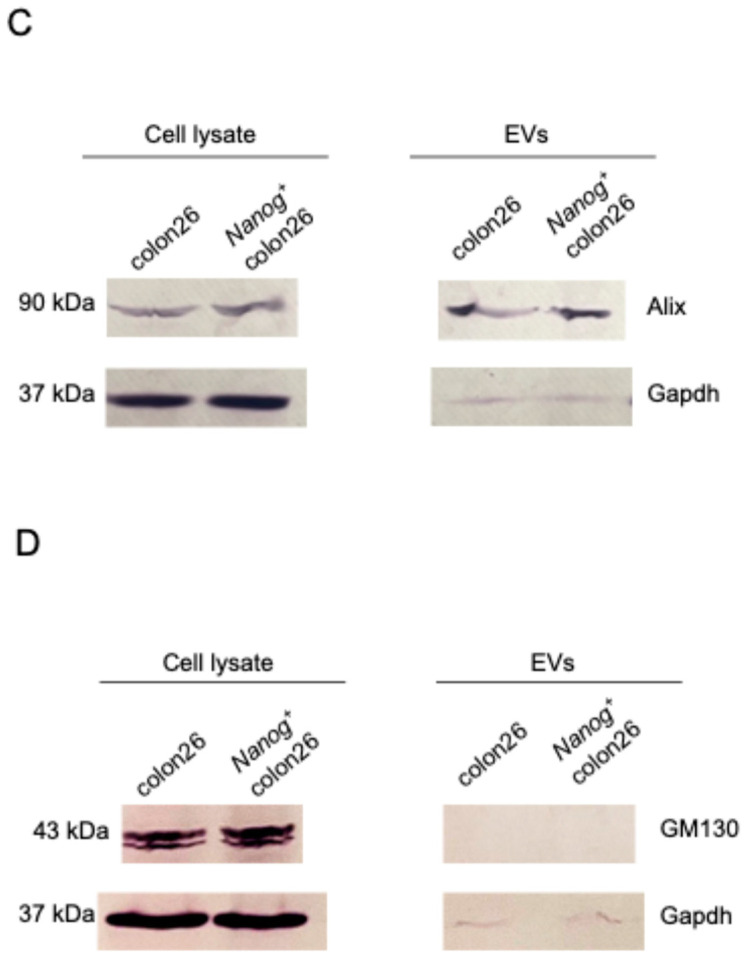
Western blotting performed to confirm the presence of *Nanog*^+^colon26EVs markers: (**A**) CD81, (**B**) TSG101, (**C**) Alix, and the absence of a negative marker, (**D**) GM130. Gapdh: loading control.

**Figure 4:**
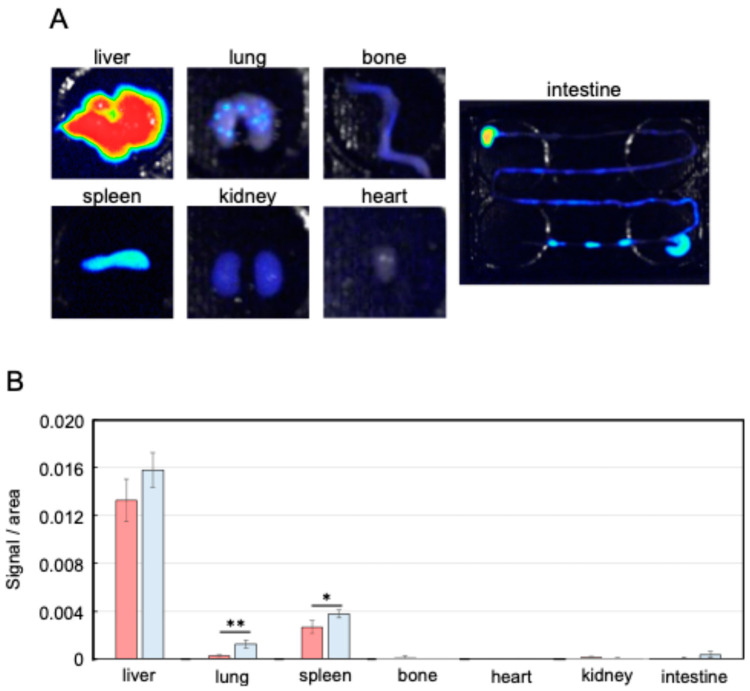
Distribution of EVs in the body. (**A**) Fluorescent images of NIR815-labeled *Nanog*^+^colon26EVs accumulated in each organ. (**B**) Accumulation of *Nanog*^+^colon26EVs (■) and colon26EVs (■) in each organ; mean ± SD for *n* = 3; ** *p* < 0.01; * *p* < 0.05.

**Figure 5:**
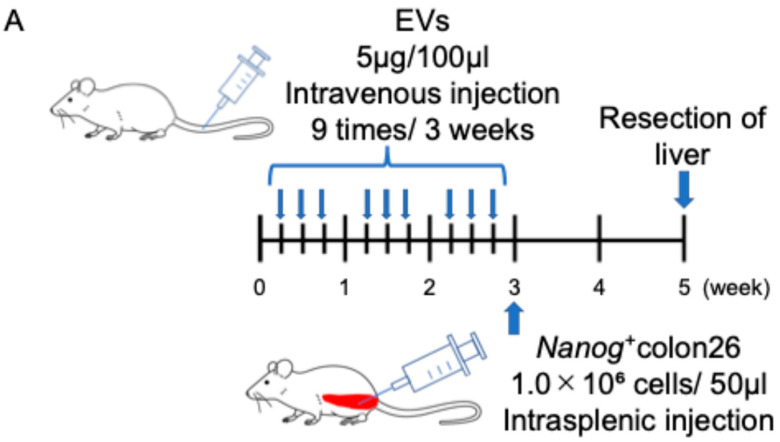

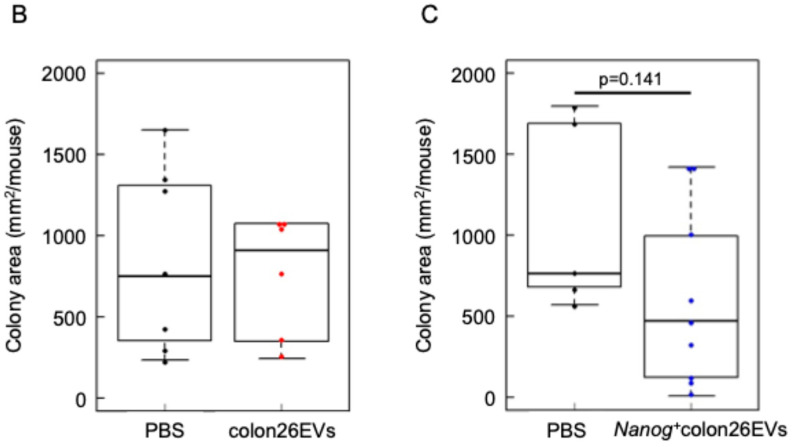
Effects of *Nanog*^+^colon26EVs on liver metastasis. (**A**) Timeline of EV and cell administrations. (**B**) Effect of colon26EVs; number of mice PBS: *n* = 7; colon26EVs: *n* = 6. (**C**) Effect of *Nanog*^+^colon26EVs; number of mice PBS *n* = 5 (2 of 7 mice died within 2 weeks after the injection of *Nanog*^+^colon26 cells); *Nanog*^+^colon26EVs: *n* = 9.

EVs isolated from *Nanog*^+^colon26 cells and colon-26 cells were confirmed by Western blotting using EVs markers such as CD81, TSG101, and Alix (Figure 3). Moreover, the absence of a negative marker, GM130, was also confirmed. Next, the circulation of EVs in the body was examined by injecting NIR815-labeled EVs (100 µL of 50 µg/mL) from the tail vein. Three hours after the injection, organs were excised for fluorescent image analysis. The liver showed the highest uptake of EVs among the seven organs (Figure 4A). In the lung and spleen, the uptake of *Nanog*^+^colon26EVs was higher than that of colon26EVs (Figure 4B). In the liver, the amount of *Nanog*^+^colon26EVs taken up tended to be greater than that of colon26EVs, although the difference was not statistically significant. Then, the effect of *Nanog*^+^colon26EVs on the metastasis of colon cells to the liver was investigated. *Nanog*^+^colon26EVs or colon26EVs were administered nine times via the tail vein at 100 µL (50 µg/mL) (Figure 5A). *Nanog*^+^colon26 cells were then injected into the spleen, and 2 weeks later, the mice were euthanized by cervical dislocation, and the livers were removed. The livers were cut into lobes and photographed. The area of tumors in the images was added up per mouse. Mice treated with colon26EVs exhibited reduced metastasis compared to those treated with PBS (Figure 5B,C). It is noteworthy that two of the mice treated with PBS died within two weeks of the injection of *Nanog*⁺colon26 cells, but all mice treated with *Nanog*⁺colon26EVs survived for two weeks.

### 2.3. Effects of Nanog^+^colon26EVs on Macrophage Phagocytic Function

Since the metastasis-suppressive effect was obtained when EVs were used as a vaccine, it was predicted that the target cells involved were immune cells. Since the involvement of macrophages has already been reported in the suppressive effect of *Nanog*^+^F10EVs on the liver metastasis of melanoma *Nanog*^+^F10 [30], we assumed that macrophages would also be involved in the case of *Nanog*^+^colon26EVs. Specifically, we focused on the phagocytic function playing a role in the elimination of unnecessary cells, including cancer cells. In the liver, EVs are primarily taken up by Kupffer cells [37], suggesting that they are likely involved in phagocytic activity. In vitro tests were performed to examine the phagocytic activity using fluorescent microbeads (MBs) as a phagocytosed model. EVs (*Nanog*^+^colon26EVs or colon26EVs) were labeled with the fluorescent dye PKH26 and used together with commercially available MBs labeled with FITC (FITC-MBs). J774.1 cells that had taken up PKH26-EVs and/or FITC-MBs were detected in the PE channel and the FITC channel, respectively, of a flow cytometer (FACSAriaII, BD Bioscience).

**Figure 6:**
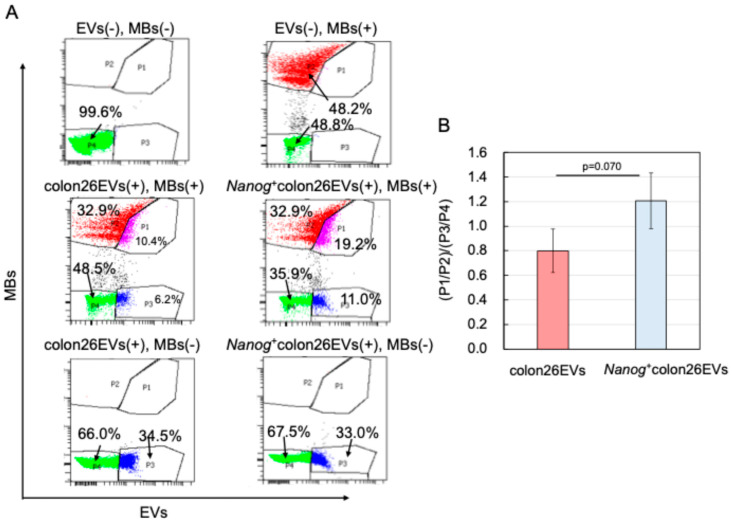

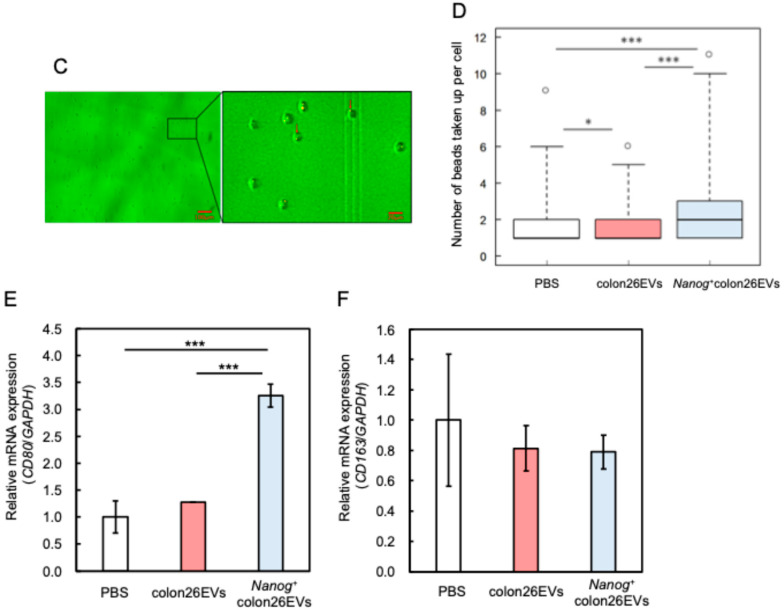
Effects of EVs on phagocytic activity of J774.1 cells. (**A**) Flow cytograms of J774.1 cells containing PKH26-EVs and/or FITC-MBs. PKH26, Ex: 551 nm, Em: 567 nm; FITC, Ex: 498 nm, Em: 517 nm. Plot colors: violet in P1, red in P2, blue in P3, green in P4. (**B**) Phagocytic activity, mean ± SD for *n* = 3. (**C**) Uptake of MBs by J774.1 single cells analyzed by fluorescent microscopy. Red arrows indicate MBs. (**D**) Number of MBs taken up per cell. °: outliers. PBS: *n* = 377, mean = 1.81, median = 1. colon26EVs: *n* = 367, mean = 1.65, median = 1. *Nanog*^+^colon26EVs: *n* = 420, mean = 2.19, median = 2. *: *p* < 0.05; ***: *p* < 0.001. (**E**) Expression of macrophage marker (*CD80*) determined by qPCR; mean ± SD for *n* = 3. ***: *p* < 0.001. (**F**) Expression of macrophage marker (*CD163*) determined by qPCR; mean ± SD for *n* = 3.

As shown in Figure 6A, a flow cytogram for a cell sample containing neither PKH26-EVs nor FITC-MBs was obtained as a double-negative control. A flow cytogram for a cell sample containing FITC-MBs alone was obtained as a PKH26-EVs negative control. From these controls, we set the P2 and P4 gates so that the fractional number of cells within the P1 and P3 gates was 0%. Figure 6 shows results obtained with cell samples containing both PKH26-EVs and FITC-MBs.

If cells that have taken up EVs have a greater tendency to take up MBs, it is expected that P1/P2 will be larger. However, a large P1/P2 ratio may also simply indicate that EVs themselves are more likely to be taken up by cells. Therefore, it is necessary to offset such an effect specific to EVs. This effect may be estimated from flow cytograms of MB negative controls. If EVs themselves are likely to be taken up by cells, it is expected that P3/P4 will be large. Therefore, we defined the effect on phagocytic activity as the net effect of EVs on the MBs uptake that may be given by [P1/P2] _[in (colon26EVs(+), MBs(+)), (*Nanog*_^+^_colon26EVs(+), MBs(+))]_/[P3/P4] _[in (colon26EVs(+), MBs(−)), (*Nanog*_^+^_colon26EVs(+), MBs(−))]_. The effect of *Nanog*^+^colon26EVs was greater than that of colon26EVs (Figure 6B).

Furthermore, the number of MBs taken up by single cells was quantified using fluorescent microscopy. After confirming the positions of J774.1 cells by a bright field image (Figure 6C), the numbers of MBs taken up into each cell were counted in the fluorescent images (Figure 6C). Compared to the control condition where PBS was added to the cell suspension, the number of MBs taken up into the cells decreased when colon26EVs were added instead of PBS, while it increased when *Nanog*^+^colon26EVs were added (Figure 6D).

### 2.4. Effects on Macrophage Polarization

It was previously reported that *CD80*-positive M1 macrophages exhibited higher phagocytic activity compared to M2 macrophages [38]. Therefore, we investigated the effect of EVs on macrophage polarization. The results demonstrated that the expression of *CD80* (a marker of M1 type) was higher in *Nanog*^+^colon26EVs than in colon26EVs (Figure 6E), whereas the expression of *CD163* (a marker of M2 type) was at the same level (Figure 6F). This suggests that *Nanog*^+^colon26EVs promoted the polarization of J774.1 cells toward the M1 phenotype and consequently contributed to the enhancement of phagocytic activity.

### 2.5. Expression of miRNAs and Their Target Genes in EVs

**Figure 7:**
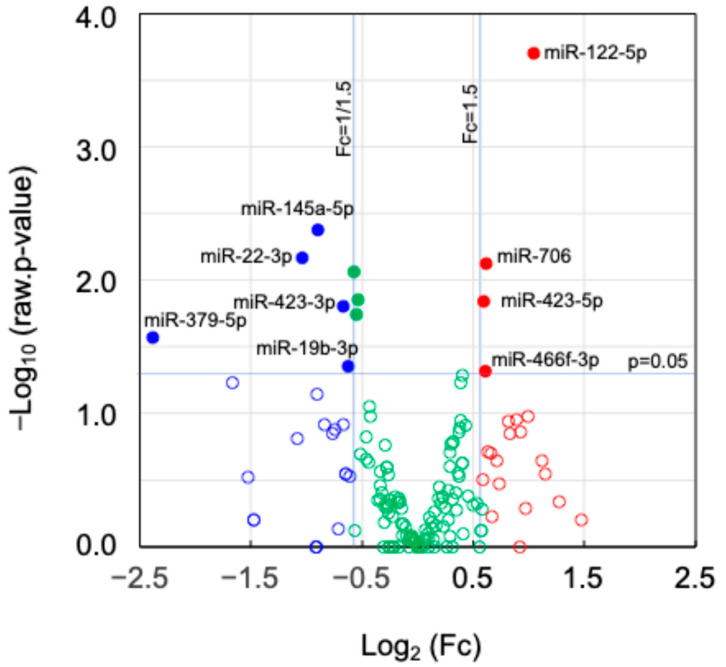
A volcano plot of miRNAs. Fold change (Fc) and p-value in statistical significance; (●) Fc ≥ 1.5 and *p* < 0.05, (●) Fc ≤ 1/1.5 and *p* < 0.05, (●) 1/1.5 < Fc < 1.5 and *p* < 0.05, (**○**) Fc ≥ 1.5 and *p* ≥ 0.05, (**○**) Fc ≤ 1/1.5 and *p* ≥ 0.05, (**○**) 1/1.5 < Fc < 1.5 and *p* ≥ 0.05, where Log_2_(1.5) = 0.585, Log_2(_1/1.5) = −0.585, −Log_10_(0.05) = 1.301.

Small RNA sequencing was conducted to find *Nanog*-dependent miRNAs that were significantly changed (Fc ≥ 1.5 or Fc ≤ 1/1.5; *p* < 0.05) in *Nanog*^+^colon26EVs as compared to colon26EVs. Nine miRNAs (up-regulated: four, down-regulated: five) were found (Figure 7), and their target genes were surveyed using TargetScanMouse8.0. Under the condition that the cumulative weighted context score was equal to or lower than −0.40, 623 genes were predicted (Table 1).

Following the prediction of 623 genes that corresponded to respective proteins, the network of those functional proteins was analyzed using STRING ver.3.10.1 and the association levels between those proteins were analyzed using cytoHubba to predict 30 genes/proteins with the highest level of association (Table 2).

### 2.6. Factors Involved in the Activation of the Immune System

Genes presumed to be closely involved in the activation of the immune system, macrophages, and phagocytic activity were searched for among 623 target genes listed in Table 1. The search was conducted in three steps. The initial step involved the selection of individual genes predicted to be most affected by *Nanog* overexpression. Specifically, the top 30 genes with the highest level of association were predicted by functional association analysis using STRING, followed by association level analysis using cytoHubba. Next, enrichment analysis was conducted to investigate the possibility that multiple gene groups may be involved in a specific pathway as a cluster and to predict dominant genes among them. The third step involved searching for genes that included the keywords “macrophage” and “phagocytosis” in their functional annotation among the 623 target genes.

#### 2.6.1. Results of the First Search

The top 30 genes predicted to be most associated with *Nanog* overexpression are shown in Table 2. The predicted effects of the top 10 genes are also listed. The top first gene, *Actb*, is an actin-related gene and is associated with cell motility and contraction. The predicted effect of the up-regulation of *Actb* is the increase in cell motility. Our in vitro tests of migration and invasion, however, did not seem to reflect the possible effect of *Actb*. The top second gene, *Ins1*, encodes insulin, a hormone that contributes to the maintenance of blood glucose levels at a proper condition. Partial suppression of *Ins1* might cause moderate hyperglycemia and contribute to metastasis suppression, as suggested by [39,40]. The top third, fourth, and sixth genes are factors promoting normal growth of brain, heart, and nerve cells, respectively. The contribution of these factors to the immune system, if any, might be minimal because they are relevant to specific organs different from the immune system. Conversely, the top 5th and 7th~10th genes are involved in the proliferation of various cells that may include immune system cells. Therefore, increased expression of these genes is desirable for increased immune system activity. The top fifth (*Grb2*) and top ninth (*Kitl*) genes, however, are down-regulated by up-regulated miR-466f-3p. Therefore, it would be effective to increase the expression of *Grb2* and *Kitl* at the genetic level in addition to increasing the expression of miR-466f-3p.

In conclusion, the predicted genes related to immune system activation by the first search are *Ins1*, *Erbb3*, *Fgfr2*, and *Esr1*.

#### 2.6.2. Results of the Second Search

Pathway enrichment analysis was conducted on a group of 623 target genes. Ten pathways in KEGG pathways and two pathways in WikiPathways were predicted to be enriched (Table 3). Two key words, “focal adhesion” and/or “PI3K-Akt(-mTOR) signaling” were found in common in the description of two of the KEGG pathways and two of the WikiPathways. Among these four pathways, WP2841 was the only one that contained both keywords in the description. Therefore, this pathway was thought to be most closely associated with *Nanog*-dependent genes. The number of genes included in WP2841 was 316, of which 24 genes were included in the 623 target genes (Table 4). Furthermore, 11 of the 24 genes were included in the top 30 of Table 2. In addition, seven of the top ten genes in Table 4 were identical to seven of the top ten genes in Table 2.

Other than these seven genes in Table 4, *Csf1r* and *Pik3cd* were of interest because of their relevance to the immune system. *Csf1r* is a macrophage colony-stimulating factor 1 receptor, which is up-regulated and is predicted to contribute to immune system activation. *Pik3cd* is an isoform of the common keyword PI3K, which mediates immune responses. Although its target is not macrophages, *Pik3cd* is involved in the development, proliferation, and migration of B cells.

In conclusion, the newly predicted genes related to immune system activation by the second search were *Csf1r* and *Pik3cd*.

#### 2.6.3. Results of the Third Search

Nine genes were selected and listed in Table 5. *Maf1*, *Ocln*, and *Ifi204* are related to macrophage differentiation, adhesion, and expansion. However, these genes are down-regulated by up-regulated miR-122-5p or miR-466f-3p. Therefore, their effects are thought to be suppressive to the immune system. With regard to phagocytosis, *Il15ra* is also down-regulated by miR-466f-3p, and therefore, its effect was thought to be suppressive to phagocytic activity.

On the other hand, *Il31*, *Csf1r*, and *Pdgfd* are predicted to be involved in the proliferation and differentiation of macrophages. Among them, *Il31* is specific to skin immunity. *Gulp1* is predicted to have a promoting effect on phagocytosis. The effects of these four genes are predicted to be promotive to the immune system because these genes are up/no-regulated by down-regulated miR-22-3p, miR-145a-5p, and miR-19b-3p.

A special remark should be made regarding *Pkm*. *Pkm* is included in the top 30 of Table 2 and its role includes the promotion of the immune checkpoint ligand PD-L1 in a STAT1 (signal transducer and activator of transcription 1)-dependent manner in macrophages lacking circadian genes, thus acting as a brake on the immune system [41]. Here, *Pkm* is down-regulated by miR-122-5p, and therefore, PD-L1 was suppressed and, consequently, immune activity was thought to be enhanced.

In conclusion, the newly predicted genes related to immune system activation by the third search were *Pdgfd*, *Gulp1*, and *Pkm* (*Csf1r* was already selected by the second search).

## 3. Material and Methods

### 3.1. Cell Culture

The mouse colon-26 cell line was obtained from RIKEN BRC and cultured in RPMI-1640 (Gibco, Thermo Fisher Scientific, Tokyo, Japan) containing 10% FBS and 1% penicillin–streptomycin at 37 °C under 5% CO_2_. When the cells reached 70–80% confluence, the medium was removed from the dish, and the cells were washed twice with PBS. Subsequently, 400 μL of a solution containing 0.25 (*w*/*v*) % trypsin and 1 mM EDTA was added to the dish. Following incubation at 37 °C under 5% CO_2_ for 5 min, the cells were detached from the dish for subculture. Mouse macrophage J774.1 was obtained from RIKEN BRC. J774.1 cells were cultured in accordance with the methodology previously described for mouse melanoma cells [25].

### 3.2. Animals

This study was conducted in accordance with the ALLIVE guidelines. The BALB/c male mice were sourced from Kiwa Laboratory Animal Research Center (Wakayama, Japan). The mice were used in the experiments following a one-week period of acclimation. The breeding room was maintained under SPF conditions with a 12 h light–dark cycle. The mice were provided with solid food (MF, Oriental Yeast Co., Ltd., Tokyo, Japan).

### 3.3. Generation of a Nanog-Overexpressing Colon Cancer Cell Line

A cell line overexpressing *Nanog* designated *Nanog*⁺colon26 was generated by introducing the pCAG-*Nanog*-IRES-puroR-EGFP vector into the colon-26 cells. The vector solution (4 µg/250 µL RPMI-1640) and Lipofectamine 2000 (Thermo Fisher Scientific) solution (5 µL/250 µL RPMI-1640) were combined and incubated at room temperature for 20 min. The mixture was added to the colon-26 culture medium and incubated at 37 °C and 5% CO_2_ for 3 h. The medium was then replaced with fresh RPMI-1640 medium, and the cells were cultured for 48 h. The *Nanog*^+^colon26 cells were then selected by culturing them in a puromycin-containing RPMI-1640 medium for 2 weeks.

### 3.4. Proliferation Activity

Cells were cultured in dishes (6 cm^ϕ^). The initial cell density was 1 × 10^5^ cells/dish. Replacing the medium with fresh medium at 48 h, the culture was continued for another 48 h. Cells were collected at 48 h and 98 h by trypsin/EDTA treatment, and the number of cells was determined.

### 3.5. Migration Activity

The migration activity of colon cells was analyzed using a Transwell^®^ 24-well plate (Corning, Corning, NY, USA). Cells were serum-starved for 24 h before use. A cell suspension (1.0 × 10^5^ cells/mL, 0.5 mL) prepared in RPMI was added to the upper Transwell insert, and 0.75 mL of RPMI medium supplemented with 10% FBS was added to the lower well and incubated for 22 h. The membrane was fixed in 100% methanol for 2 min, stained with hematoxylin for 2 min, stained with eosin for 2 min, and photographed.

### 3.6. Invasion Activity

The invasion activity of colon cells was analyzed by culture on Matrigel^TM^Matrix Basement Membrane (BD Biosciences, Tokyo, Japan) that was coated on a porous membrane of a Transwell^®^ 24-well plate. The number of cells that passed through the Matrigel and porous membrane for 22 h was analyzed in the same manner as above (Section 3.5).

### 3.7. Gelatine Zymography for Matrix Metalloproteinase (MMP) Activity

Protein samples for zymography were prepared from colon cells after the cells were incubated in the RPMI medium (pH 5.5) for 3 h at 25 °C to maintain enzymatic activity. A sample solution containing 10 μg protein was separated by SDS-PAGE. After SDS-PAGE, the gel was washed with a washing buffer (pH 7.5) containing 0.05 M Tris-HCl, 2.5 *v*/*v*% TritonX-100, 5 mM CaCl_2_, 1 μM ZnCl_2_, and 0.016% NaN_3_, for 30 min. The gel was stained with CBB and decolorized with a methanol–acetic acid solution. Band images were quantified using ImageJ software (Version 1.54k).

### 3.8. Quantification of Colon Metastasis to Liver

To quantify the liver metastasis, colon-26 cells or *Nano*g^+^colon26 cells (1.0 × 10⁶ cells/50 µL PBS) were injected into the spleen of 8~9-week-old mice. Two weeks following the injection of cells, the mice were euthanized by cervical dislocation, and the livers were removed (Figure 2A). The livers were sectioned into lobes and photographed. The tumors in the images were made black using the Image J software, and the surface area of the tumors was calculated (Figure 2B).

### 3.9. Preparation of EVs

EV-depleted FBS was prepared from FBS by centrifugation at 100,000× *g* at 4 °C for a total of 80 min. The cells were cultured in RPMI-1640 medium for 48 h, after which the medium was removed, and the cells were washed with PBS. Following a 72 h incubation period in EV-depleted medium (RPMI-1640, 10% EV-depleted FBS, 1% penicillin–streptomycin), the culture supernatant was subjected to centrifugation at 2000× *g* at 4 °C for 20 min. Subsequently, the supernatant was centrifuged at 10,000× *g* at 4 °C for 40 min, after which the resulting supernatant was centrifuged again at 120,000× *g* at 4 °C for 80 min. The precipitate was suspended in 10 mL of phosphate-buffered saline (PBS) and filtered through a 0.22 µm pore-size filter. The filtrate was divided into two fractions in a volume ratio of 1:9. The larger fraction was then centrifuged again at 120,000× *g* at 4 °C for 80 min, after which the precipitate was suspended in PBS. The resulting solution contained mainly exosomes and some portion of larger particles, and therefore, we called this an EV solution. The remaining fraction was combined with 50 µL of RIPA buffer (25 mM Tris-HCl, 150 mM NaCl, 1% NP-40, 1% sodium deoxycholate, 0.1% SDS, pH 7.6; Thermo Fisher Scientific) and employed as a protein sample for the BCA (bicinchoninic acid) assay (Pierce^®^ BCA TM Protein Assay kit, Thermo Fisher Scientific). The mode diameter and mean diameter of EVs were approximately 90 nm and 130 nm, respectively. The presence of exosomes was confirmed by the detection of marker proteins for exosomes, CD81, TSG101, and Alix, by Western blot analysis. The absence of contamination of cellular components was checked by the absence of GM130.

### 3.10. Western Analysis of EVs Markers, Negative Marker, and Gapdh

A protein sample of cells was prepared as follows: Following the rinsing of 70–80% confluent cells with PBS, a RIPA buffer was added to the culture dish. The culture dish was then placed on ice for a period of 15 min. Subsequently, the cells were detached from the culture dish with a cell scraper and collected in a 1.5 mL microtube. The cell suspension was subjected to sonication on ice and centrifugation at 20,000× *g* at 4 °C for 15 min. The supernatant was collected as a protein sample of the cells. A protein solution of EVs was prepared as follows: The pellet of EVs obtained as the precipitate of ultracentrifugation was suspended in a RIPA buffer and allowed to stand on ice for 15 min. The protein concentration was determined by BCA assay. A protein solution was prepared by mixing a 1/6 volume of a 0.375 M Tris-HCl (pH 6.8) buffer solution containing 93 μg/mL DTT, 0.12 g/mL SDS, 0.6 mL/mL glycerol, and 0.6 mL/mL bromophenol blue. Subsequently, the solution was heated to 95 °C for 5 min and applied to SDS-PAGE at 150 V. Blotting onto a PVDF membrane was conducted at 100 V for 3 h at 4 °C. Subsequently, the PVDF membrane was immersed in a TBS-T solution (Tris-buffered saline (25 mM Tris, pH 7.4, 150 mM NaCl) containing 1 (*v*/*v*) % Tween 20) containing 5 (*w*/*v*) % skim milk at 25 °C for 30 min. Subsequently, the PVDF membrane was incubated in a 5% skim milk TBS-T solution containing primary antibody at 25 °C for 3 h. The primary antibodies used were mouse anti-mouse Gapdh (1:1000, sc-32233; Santa Cruz Biotechnology, Dallas, TX, USA), mouse anti-mouse CD81 (1:500, sc-166029; Santa Cruz Biotechnology, Dallas, TX, USA), mouse anti-mouse TSG101 (1:500, sc-7964; Santa Cruz Biotechnology, Dallas, TX, USA), mouse anti-mouse Alix (1:500, sc-53538; Santa Cruz Biotechnology, Dallas, TX, USA), and mouse anti-mouse GM130 (#610822; BD Bioscience), respectively. Following three washes with TBS-T, the membrane was incubated in TBS-T containing a secondary antibody (anti-mouse immunoglobulin conjugated to alkaline phosphatase, Promega, Madison, WI, USA) at 25 °C for 1 h. Membranes were washed three times with TBS-T and then incubated with Western Blue Stabilized Substrate (Promega) for alkaline phosphatase at 25 °C.

### 3.11. Analysis of the Transfer of EVs to Various Organs in Mice

EVs were stained with CellVueTM NIR815 (Invitrogen, Thermo Fisher Scientific) and introduced into mice by tail vein injection at a dose of 5 µg per 100 µL of PBS. The mice were euthanized at 3 h and various organs were extracted for imaging analysis with the Pearl Trilogy imaging system (LI-COR) at 800 nm. The integrated fluorescence intensity of the entire organ was divided by the organ area (signal/area) in order to estimate the density of EVs accumulated in each organ.

### 3.12. In Vivo Test of the Effects of EVs on Metastasis

The introduction of EVs into mice was conducted via tail vein injection at a dose of 5 µg/100 µL PBS, administered three times per week for a period of three weeks (Figure 2C). Subsequently, *Nanog*⁺colon26 cells (1.0 × 10⁶ cells/50 µL PBS) were injected into the spleen of the mice. Two weeks later, the mice were euthanized by cervical dislocation, and the livers were removed. The quantification of colon metastasis was conducted according to the method described above (Section 3.8).

### 3.13. Phagocytic Activity Test

The phagocytic activity of J774.1 cells was estimated by means of a microbeads (MBs) uptake test. EVs were stained with a fluorescent dye, PKH26 (Ex: 551 nm, Em: 567 nm, DOJINDO). FITC-labeled microbeads (FITC-MBs) (Ex: 441 nm, Em: 486 nm, Sigma, Kanagawa, Japan) were incubated at 37 °C for 1 h in RPMI medium containing 10% heat-inactivated FBS for opsonization. J774.1 cells were incubated in RPMI medium with or without FITC-MBs and/or PKH26-EVs for a period of 2 h. Following this incubation period, the J774.1 cells were collected for flow cytometry analysis using the FACSAriaII (BD Bioscience). Phagocytic activity was also analyzed at the single-cell level by fluorescent microscopy. In this case, EVs were used without fluorescent labeling. The cell suspension was then placed in a hemocytometer, and bright field and fluorescent images were taken with a fluorescent microscope. The bright field and fluorescent images were merged using ImageJ to determine the percentage of cells that had taken up MBs and the number of MBs taken up by each individual cell. Three wells were prepared for PBS, colon26EVs, and *Nanog*^+^colon26EVs groups and 200 to 300 cells were placed in each well.

### 3.14. Analysis of miRNAs and Their Target Genes

The differential expression analysis of miRNAs expressed in *Nanog*^+^colon26EVs and colon26EVs was outsourced to Macrogen Japan (Tokyo, Japan). The magnitude of variation (fold change, Fc) and the statistical significance (*p*-value) of the respective miRNA components were obtained.

### 3.15. Statistical Analysis

Each test sample was subjected to two or three analyses, with the average of the two or three results recorded as the value for one test sample. The results are presented as the mean and standard deviation (SD) for the number of samples (n). The results of the metastatic colony analyses are presented in box plots. Outliers depicted in box plots were identified through the application of a Smirnoff–Grubbs test, which determined that they exhibited a probability of greater than 0.05. The statistical significance between two specific data groups was analyzed by a two-tailed Student’s *t*-test. The statistical significance of the results is indicated by a *p*-value or by the use of asterisks; ***: *p* < 0.001; **: *p* < 0.01; *: *p* < 0.05.

## 4. Discussion

This study consists of two propositions. First, do cancer cells become more malignant due to *Nanog*? Second, do EVs obtained from *Nanog* overexpressing cancer cells suppress cancer metastasis? In *Nanog*^+^colon26 cells, the answer to the first proposition was not necessarily yes. In other words, the properties of the cells in vitro did not show any results indicating high malignancy as cancer cells, except for an increased proliferation rate. However, the metastatic ability in vivo was enhanced, suggesting a more malignant property in cancer cells. The answer to the second proposition was yes. We thought that the essential effect of *Nanog* was to enhance the undifferentiated state of cells rather than to make cells more malignant. iPS cells are highly undifferentiated cells. That EVs derived from iPS cells showed a high metastasis suppression effect supports this idea [31].

Regarding the action mechanism of EVs, we focused on the involvement of macrophages. Phagocytosis activity tests showed that the activity of *Nanog*^+^colon26EVs was higher than that of colon26EVs. Furthermore, it was estimated that the antitumor ability of macrophages was enhanced by the enhanced expression of the M1 marker *CD80*. Then, we conducted small RNA sequencing to predict miRNAs and target genes involved in macrophage activation. As a result, nine candidate genes were predicted (Table 6). Based on these results, we plan to conduct the following experiments. The first is control at the miRNA level, which is an increase in miR-122-5p and/or miR-466f-3p. *Pkm* and *Ins1* are down-regulated by miR-122-5p and miR-466f-3p, respectively. A further increase in miR-122-5p and/or miR-466f-3p will increase the *Pkm*- and *Ins1*-dependent suppression effects. On the other hand, the other seven genes in Table 6 are regulated by miR-22-3p, miR-145a-5p, miR-423-3p, or miR-19b-3p. Metastasis suppressing effects obtained via these four miRNAs and seven target genes may be enhanced by further reducing miRNAs expression levels or by increasing the expression levels of seven target genes. We plan to prioritize experiments starting with genes that are thought to be more closely related to macrophages, i.e., *Pdgfd*, *Gulp1*, and *Csf1r*. Next is *Pik3cd*, which is related to B-cell activation, followed by *Erbb3*, *Fgfr2*, and *Esr1*, which are related to cell proliferation and differentiation of immune system cells.

At present, it is still difficult to discuss commonalities and characteristics between types of cancer. The limitation of this research is that the molecular mechanism of the role of *Nanog* is still largely unknown. It is important to focus on specific cancers and analyze their signaling pathways even if the clarified pathways may be fragmentary. Furthermore, it is more important to consider the issues of side effects. We have not yet gained any knowledge about these issues. Unless these problems are resolved, we will not be able to overcome our limitations. However, the findings of this study certainly support our research in a promising direction.

## 5. Conclusions

*Nanog* overexpression enhanced the metastatic potential of colon-26 cells, though in vitro properties of *Nanog*^+^colon26 were not so malignant as expected. *Nanog*^+^colon26EVs were effective for the suppression of metastasis of *Nanog*^+^colon26 to the liver. The involvement of macrophages in the suppressive effects was demonstrated. The up-regulation of two miRNAs and seven genes and the down-regulation of four miRNAs and two genes were predicted to be effective for the suppressive effects.

## Figures and Tables

**Table 1:** Number of up-regulated and down-regulated miRNAs in *Nanog*^+^colon26EVs. Total number of predicted target genes with high efficiency, and number of genes among them predicted as top 30 genes with highest association.

	miRNA	Log_2_(Fc)	Number of Target Genes		
			*p* < 0.05 CWCS ≤ −0.40		Targeted by Each miRNA Among Top30 Genes
*Nanog*^+^colon26EVs/ colon26EVs	miR-122-5p	1.04	29	321	2
	miR-706	0.62	62		3
	miR-466f-3p	0.61	146		6
	miR-423-5p	0.59	84		2
	miR-379-5p	−2.38	7	302	0
	miR-22-3p	−1.04	103		8
	miR-145a-5p	−0.89	48		4
	miR-423-3p	−0.67	8		1
	miR-19b-3p	−0.62	136		4
			Sum	623	30

**Table 2:** Top 30 genes with highest association level predicted by STRING and cytoHubba. miRNAs shown in red and blue are those up-regulated and down-regulated, respectively. Predicted effects are described for top 10 genes. Arrow notations indicate up- or no-regulation (→up/no) and down-regulation (→down) of target genes.

**Rank**	**Gene**	**miRNA**	**Predicted Effects**	**Rank**	**Gene**	**miRNA**	**Rank**	**Gene**	**miRNA**
1	*Actb*	miR-145a-5p	cell motility and contraction→up/no	11	*Csf1r*	miR-22-3p	21	*Mecp2*	miR-22-3p
2	*Ins1*	miR-466f-3p	blood glucose decrease→down	12	*Sirt1*	miR-22-3p	22	*Mlxipl*	miR-706
3	*Fgf17*	miR-423-5p	normal brain development→down	13	*H3f3b*	miR-22-3p	23	*Socs1*	miR-19b-3p
4	*Fgf16*	miR-466f-3p	normal heart development→down	14	*Angpt2*	miR-145a-5p	24	*Hnrnph2*	miR-145a-5p
5	*Grb2*	miR-466f-3p	linking of cell growth factor receptor and Ras signaling→down→growth inhibition	15	*Cdkn1b*	miR-706	25	*Pkm*	miR-122-5p
6	*Ngf*	miR-423-5p	normal nerve growth→down	16	*Ppara*	miR-22-3p	26	*Prkcd*	miR-466f-3p
7	*Erbb3*	miR-22-3p	development of various organs and myeloid cell differentiation→up/no	17	*Slc2a4*	miR-466f-3p	27	*Arrb1*	miR-22-3p
8	*Fgfr2*	miR-423-3p	cell proliferation, differentiation, migration, apoptosis→up/no	18	*Mycn*	miR-19b-3p	28	*Sgk1*	miR-19b-3p
9	*Kitl*	miR-466f-3p	cell survival, proliferation, stem cell maintenance→down	19	*Ccnd2*	miR-19b-3p	29	*Hnrnpf*	miR-706
10	*Esr1*	miR-22-3p	cellular proliferation and differentiation→up/no	20	*G6pc3*	miR-122-5p	30	*Actg1*	miR-145a-5p

**Table 3:** Pathways predicted to be enriched in 623 target genes/proteins.

Pathway		Description	Count in Network	Strength
KE	mmu04010	MAPK signaling pathway	20 of 287	0.40
	mmu04014	Ras signaling pathway	17 of 225	0.44
	mmu04015	Rap1 signaling pathway	18 of 208	0.50
	mmu04068	FoxO signaling pathway	15 of 129	0.63
	**mmu04151**	PI3K-Akt signaling pathway	23 of 353	0.38
	mmu04152	AMPK signaling pathway	11 of 124	0.51
	**mmu04510**	Focal adhesion	17 of 225	0.45
	mmu04930	Type II diabetes mellitus	7 of 48	0.73
	mmu04931	Insulin resistance	10 of 109	0.52
	mmu04960	Aldosterone-regulated sodium reabsorption	6 of 38	0.76
WK	**WP2841**	**Focal adhesion: PI3K-Akt-mTOR signaling pathway**	**24 of 316**	**0.44**
	**WP85**	Focal adhesion	15 of 185	0.47

Focal adhesion is a common keyword in three pathways (bold in the pathway column), and PI3K-Akt(-mTOR) is a common keyword in two pathways (bold). Among four pathways, WP2841 contains these two keywords (bold in the description column).

**Table 4:** Genes included in the 316 genes that make up pathway WP2841 among the 623 target genes listed in Table 1. Genes shown in bold are included in the top 30 (Table 2). miRNAs shown in red and blue are those that are up-regulated and down-regulated, respectively.

Rank	Name	Score	miRNA	Rank	Name	Score	miRNA	Rank	Name	Score	miRNA
1	***Ins1***	1500	miR-466f-3p	9	***Angpt2***	48	miR-145a-5p	15	*Rab14*	2	miR-466f-3p
2	***Fgf17***	1490	miR-423-5p	10	*Pik3cd*	6	miR-22-3p	15	*Efna3*	2	miR-423-5p
3	***Fgf16***	1488	miR-466f-3p	11	***Slc2a4***	5	miR-466f-3p	15	*Pfkfb2*	2	miR-466f-3p
4	***Grb2***	1450	miR-466f-3p	11	*Pdpk1*	5	miR-466f-3p	15	*Fgf12*	2	miR-706
5	***Kitl***	1440	miR-466f-3p	13	***Cdkn1b***	4	miR-706	21	*Gys1*	1	miR-122-5p
5	***Ngf***	1440	miR-423-5p	13	*Pdgfd*	4	miR-145a-5p	21	*Ppp2r5e*	1	miR-19b-3p
7	***Fgfr2***	751	miR-423-3p	15	*Ddit4*	2	miR-423-5p	21	*Itgb8*	1	miR-145a-5p
8	***Csf1r***	749	miR-22-3p	15	*Rab10*	2	miR-706	21	*Thbs3*	1	miR-423-5p

**Table 5:** Genes relevant to the functions of macrophages and phagocytosis. miRNAs shown in red and blue are those that are up-regulated and down-regulated, respectively. Arrow notations indicate the up/no-regulation (→up/no) and down-regulation (→down) of target genes.

Keyword	Gene	miRNA	Predicted Effects
Macrophage	*Maf1*	miR-122-5p	Required for monocytic, macrophage, osteoclast, and islet beta cell differentiation.→down
	***Pkm***	miR-122-5p	Promotes in a STAT1-dependent manner, the expression of the immune checkpoint protein PD-L1 in ARNTL/BMAL1-deficient macrophages, consequently suppresses immune activity.→down
	*Ocln*	miR-122-5p	Regulation of the tight junction and macrophage adhesion and spreading →down
	*Ifi204*	miR-466f-3p	Inhibits cell growth and may be involved in macrophage differentiation.→down
	*Il31*	miR-22-3p	May function in skin immunity, enhances myeloid progenitor cell survival in vitro, and induces amyloid A protein expression in macrophages.→up/no
	*Csf1r*	miR-22-3p	Regulation of survival, proliferation and differentiation of macrophages and monocytes and plays an important role in innate immunity and in inflammatory processes.→up/no
	***Pdgfd***	miR-145a-5p	Plays an important role in wound healing and Induces macrophage recruitment.→up/no
Phagocytosis	*Il15ra*	miR-466f-3p	Required for IL15-induced phagocytosis in neutrophils→down
	***Gulp1***	miR-19b-3p	Required for efficient phagocytosis of apoptotic cells.→up/no

Genes in bold are high priority for further analysis in the next research phase.

**Table 6:** Predicted genes contributing to promoting and activating macrophages and the immune system. Up-regulated and down-regulated miRNAs in *Nanog*^+^colon26 are indicated in red and blue, respectively.

miRNAs	Genes	Most Relevant Keyword
miR-122-5p	*Pkm*	Immune checkpoint
miR-466f-3p	*Ins1*	Pre-diabetes condition
miR-22-3p	*Erbb3*	Cell growth and differentiation
	*Esr1*	
	*Csf1r*	
	*Pik3cd*	B-cell
miR-145a-5p	*Pdgfd*	Macrophage
miR-423-3p	*Fgfr2*	Cell growth and differentiation
miR-19b-3p	*Gulp1*	Phagocyte

## Data Availability

The datasets generated and/or analyzed during the current study are available from the corresponding author on reasonable request.
